# Insights from Microbial Transition State Theory on Monod’s Affinity Constant

**DOI:** 10.1038/s41598-020-62213-6

**Published:** 2020-03-24

**Authors:** Pablo Ugalde-Salas, Elie Desmond-Le Quéméner, Jérôme Harmand, Alain Rapaport, Théodore Bouchez

**Affiliations:** 1 INRAE, Univ Montpellier, LBE, 102 avenue des Etangs, 11100 Narbonne, France; 2 MISTEA, INRAE, Univ. Montpellier, Montpellier SupAgro, Montpellier, France; 3 Université Paris-Saclay, INRAE, UR PROSE, 92160 Antony, France

**Keywords:** Microbial ecology, Cellular microbiology

## Abstract

Microbial transition state theory (MTS) offers a theoretically explicit mathematical model for substrate limited microbial growth. By considering a first order approximation of the MTS equation one recovers the well-known Monod’s expression for growth, which was regarded as a purely empirical function. The harvest volume of a cell as defined in MTS theory can then be related to the affinity concept, giving a new physical interpretation to it, and a new way to determine its value. Consequences of such a relationship are discussed.

## Introduction

Since the success of Monod’s expression (Equation 1) to model substrate-limited microbial growth^1^, many expressions have been proposed^2^, accounting for a range of phenomena including substrate inhibition and population density effects^3,4^. All of these expressions rely on empirical rules, differently to enzymology for which analogues of Monod and Haldane expressions have been mathematically derived^5^). Microbial transition state theory^6^ recently introduced a new expression for microbial growth based on the statistics of molecules distribution in a medium inspired from chemical transition state theory. In this communication we explore the physical meaning of the affinity concept through the lens of MTS theory and particularly show how it may provide a novel interpretation of Monod’s growth function.

1 $${\mu }_{max}\frac{s}{{{\rm{K}}}_{{\rm{s}}}+{\rm{s}}}$$

Equation 1 represents the Monod growth function, where ${\mu }_{max}[1/day]$ is the maximal growth rate, ${\rm{s}}[g/L]$ represents the substrate concentration of the medium, and ${{\rm{K}}}_{{\rm{s}}}[g/L]$ is known as the “affinity constant”. Earlier works on kinetics^7,8^ show the differences in reported literature values for the affinity constant for the same species: these differences are explained by culture history, quality of the experimental data, and posterior data analysis. However little to no consensus can be found in the literature on its interpretation. Furthermore in a review of theoretical derivations of the Monod growth function^9^ the author concludes that no clear interpretation may be given to the affinity constant. A revision of the affinity concept in Microbiology was made by Button^10^, where fourteen different expressions for affinity are documented. The concept is largely influenced by the Michaelis-Menten model for enzyme kinetics interpretation of affinity from receptor and ligand binding sites, since Monod’s expression for growth is mathematically equivalent to the Michaelis-Menten expression. As stated by Monod himself, Monod’s growth function is purely empirical, while Michaelis-Menten expression has a rigorous theoretical justification^5^, thus one might wonder if the concept of affinity for representing cell growth has a solid conceptual ground.

MTS theory relates the growth rate to the amount of energy available to perform cellular work. The central idea of bioenergetics is that the energy consuming anabolism can only be thermodynamically feasible if it is coupled with an energy yielding catabolism. The overall reaction resulting from the coupling is known as metabolism^11^. The formulation and complexity of both catabolism and anabolism vary greatly depending on the objective the modeller has in mind. On the one hand, when describing the metabolic pathways within a specific microbial species, the formulation takes into account ATP formation and intra cellular intermediates and quickly becomes a very complex web, e.g.^12^. On the other hand if one is interested in observing the general metabolism of a culture at a macroscopic level then the situation simplifies to just a couple of reactions^11^. We will focus on the latter.

Let us consider a first reaction representing catabolism (Eq. 2), a second reaction representing anabolism (Eq. 3), then a linear combination of the two creates metabolism (Eq. 4): by completing *λ* times the catabolism the energy requirements of the global metabolic reaction are fulfilled^13^ (its negative free enthalpy constitutes the driving force for growth).

2 $${E}_{d}+a{E}_{a}\to bP;\,{\Delta }_{{\rm{r}}}{G}_{cat} < 0\backslash \cdot \lambda  > 0$$

3 $$dP\to {B}_{x}+c{E}_{a};\,{\Delta }_{{\rm{r}}}{G}_{an} > 0$$

4 $$\lambda {E}_{d}+(\lambda a-c){E}_{a}\to {B}_{x}+(\lambda b-d)P;\,{\Delta }_{r}{G}_{met}=\lambda {\Delta }_{r}{G}_{cat}+{\Delta }_{r}{G}_{an} < 0$$

where ${E}_{d}$, ${E}_{a}$, and *P* stand for electron donor, electron acceptor, and products, respectively. ${B}_{x}$ represents an equivalent biomass unit, for instance ${B}_{x}=C{H}_{1.8}{O}_{0.5}{N}_{0.2}$ is a generic composition of one C-mole of biomass^14^. $a,b,c,d$ are stoichiometric coefficients. Finally ${\Delta }_{r}G$ represents the Gibbs free energy variation for each reaction.

The reader should notice that $\lambda$ is the inverse of the yield as usually expressed (*y*_x/s_) in microbiology as shown in the Eq. 5.

5 $${y}_{x/s}=\frac{1}{{y}_{s/x}\,}=\frac{1}{\lambda }$$

${y}_{x/s}$ represents how many moles of biomass are formed per mole of substrate consumed, conversely ${y}_{s/x}=\lambda$ represents how many moles of substrate are being consumed per mole of biomass formed. The methods reviewed by Kleerebezem *et al*.^11^ allow computing $\lambda$ from mass balanced reactions with examples coming from a variety of biological process.

MTS theory demonstrates on a theoretically explicit ground a growth rate expression $\mu$ of a culture of bacteria limited by an electron donor in perfectly mixed conditions^6^. More precisely, if we denote by $s$ the concentration of the limiting electron donor and $x$ the concentration of the species then these two concentrations are dynamically related by:

6 $$\dot{x}=\mu (s)x={\mu }_{max}\exp \left(\frac{-\lambda }{{V}_{h}\,s}\right)x$$

where ${V}_{h}$, known as the harvest volume, represents the volume to which each microbe has access in order to harvest the substrate $s$ during the time between two cell divisions. It is worth pointing out that the harvest volume is an average characteristic.

If one considers a first order approximation of the exponential function near zero (see supplementary material) then one recovers Monod’s expression of growth:

7 $${\mu }_{max}{e}^{-\frac{\lambda }{{V}_{h}\cdot s}}\approx {\mu }_{max}\frac{s}{s+\frac{\lambda }{{V}_{h}}}={\mu }_{max}\frac{s}{s+\frac{1}{{y}_{x/s}\,{V}_{h}}}$$

**Figure 1:**
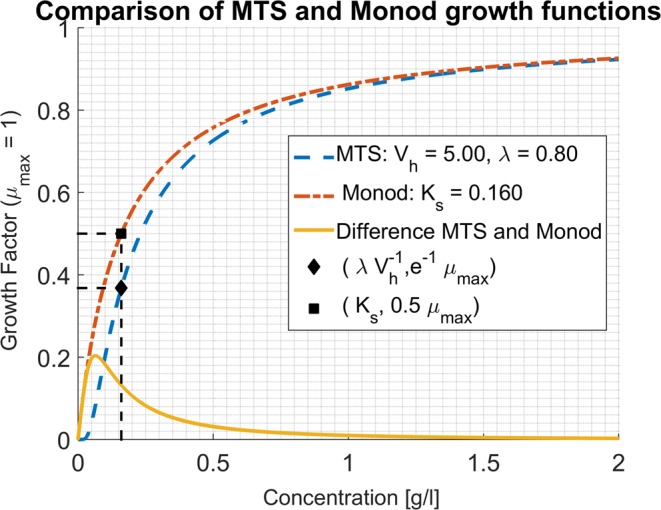
Example of plots of Eqs. 1 and 6, with the values chosen such that ${{\boldsymbol{K}}}_{{\boldsymbol{s}}}=\frac{{\boldsymbol{\lambda }}}{{{\boldsymbol{V}}}_{{\boldsymbol{h}}}}$. The measurement of the harvest volume from growth experiments can be obtained in an analogous fashion to the determination of the affinity constant: by noting ${{\bf{s}}}^{\ast }$ the value of substrate concentration at which the growth rate is ${{\boldsymbol{e}}}^{-1}{{\boldsymbol{\mu }}}_{{\boldsymbol{\max }}}$ (represented by the black diamond) one obtains ${{\boldsymbol{V}}}_{{\boldsymbol{h}}}$ by the formula ${{\boldsymbol{V}}}_{{\boldsymbol{h}}}=\frac{1}{{{\boldsymbol{y}}}_{{\boldsymbol{x}}/{\boldsymbol{s}}}\,{{\boldsymbol{s}}}^{\ast }}$, similarly to the ${{\boldsymbol{K}}}_{{\boldsymbol{s}}}$ value identified as the concentration for which the specific growth rate ${\boldsymbol{\mu }}$ is equal to $\frac{{{\boldsymbol{\mu }}}_{{\boldsymbol{\max }}}}{2}$ in the Monod expression, (represented by the black square).

The approximation holds true for high substrate concentrations. More precisely, it can be shown that the two curves differ by less than 10% for $s\ge 1.92{K}_{s}$, (see supplementary material). In Fig. 1 the graphical comparison of both growth functions can be seen for a given set of parameters. The MTS growth function is approximated very well by the Monod growth function, which is reassuring from a practical point of view: in a re-examination of the kinetics of *Escherichia coli*^15^ different empirical substrate limiting expressions- all of them with a Monod-like shape- were compared and no difference was found in the identifiability of their parameters.

Note also that in Eq. 7, $\frac{1}{{y}_{x/s}\,{V}_{h}}$ replaces the ${K}_{s}$ parameter of Monod’s expression. In that sense the affinity constant can be interpreted as a decreasing function of the harvest volume of the cell and its yield per mole of substrate. On one hand, associating low ${{\rm{K}}}_{{\rm{s}}}$values to large harvest volumes is well in line with our understanding of the affinity concept, since a cell that can harvest substrate molecules in a more extended region should be less substrate limited. On the other hand, the fact that a low ${K}_{s}$ value could be due to a higher conversion yield of substrate to biomass sheds a new light on the affinity concept. The order of magnitude of ${V}_{h}$ can be seen from Table 1 for some literature references for *E. Coli* ML 30. In the cases where no yield was reported the energy dissipation method^11^ can be used as illustrated in Table 1 and supplementary material. For computing the yield a unique biomass formula was used ($C{H}_{1.8}{O}_{0.5}{N}_{0.2}$). However, for each case, the biomass composition could be different and, consequently, the yield, thus contributing to the explanation of the observed variability of ${{\rm{K}}}_{{\rm{s}}}$.

**Table 1:** Literature values (Table 2^8^) of *K*_*s*_ and calculation of ${{V}}_{{h}}=\frac{{\lambda }}{{{K}}_{{s}}}$, for different chemostat experiments using hexoses as substrates.

*K*_*s*_ reported [${\boldsymbol{\mu }}{\boldsymbol{g}}{\boldsymbol{/}}{\boldsymbol{L}}$] for E. Coli ML 30	*λ* (gS/gX) *Estimated by Energy dissipation Method (Supplementary Material) **Measured during experiment	*V*_*h*_[*L*/*gX*]	*V*_*h*_[μm^3^/cell] (cell dry weight: 2.8 10^−13^gr/cell) (Ref: ^16^ BNID: 103904 Neidhart *et al*.)	Radius $[{\boldsymbol{\mu }}{\boldsymbol{m}}]$ of a sphere of volume ${V}_{h}$
33 (Ref: ^17^)	${\lambda }^{\ast }=1.89$	$4.91\cdot {10}^{4}$	$1.6\cdot {10}^{7}$	156
33 (Ref: ^17^)	${\lambda }^{\ast }=1.88$	$4.85\cdot {10}^{4}$	$1.6\cdot {10}^{7}$	156
53 (Ref: ^15^)	${\lambda }^{\ast }=1.88$	$3.02\cdot {10}^{4}$	$9.94\cdot {10}^{6}$	133
72 (Ref: ^15^)	${\lambda }^{\ast }=1.88$	$2.22\cdot {10}^{4}$	$7.32\cdot {10}^{6}$	120
76 (Ref: ^18^)	${\lambda }^{\ast \ast }=2.22$	$2.92\cdot {10}^{4}$	$8.19\cdot {10}^{6}$	125
90 (Ref: ^18^)	${\lambda }^{\ast \ast }=2.22$	$2.47\cdot {10}^{4}$	$6.91\cdot {10}^{6}$	118
100 (Ref: ^18^)	${\lambda }^{\ast \ast }=2.22$	$2.22\cdot {10}^{4}$	$6.22\cdot {10}^{6}$	114
132 (Ref: ^18^)	${\lambda }^{\ast \ast }=2.22$	$1.68\cdot {10}^{4}$	$4.71\cdot {10}^{6}$	104
125 (Ref: ^18^)	${\lambda }^{\ast \ast }=2.22$	$1.77\cdot {10}^{4}$	$4.98\cdot {10}^{6}$	105

On a more conceptual ground, the MTS approach proposes a way to revisit our current perception of the “affinity-concept” of a microbial culture for a given substrate. It offers an alternative view of the microbial affinity notion than its enzymatic analogue related to Michaelis-Menten theory. It unravels a contribution that is related to the yield (mole of biomass formed per mole of substrate consumed) from another that represents the capacity of the microbial culture to explore a fraction of its surroundings in order to harvest substrate (${V}_{h}$ term). To this extent, it allows to compute the affinity constant from the knowledge of the yield and the harvest volume, which is a completely new approach to determining this constant.

This analysis thus plants a seed towards a more physically grounded view of affinity than earlier proposals made from attempts to theoretically derive Monod’s equation^9^. The physical interpretation of the affinity concept raises new opportunities to analyse and experimentally challenge the meaning of the ${V}_{h}$ parameter. Particularly interesting would be to assess to which extent ${V}_{h}$ constitutes an intrinsic trait of the microbial culture, or if extrinsic attributes associated to the culture conditions (such as agitation, viscosity or ionic force) could also significantly influence its value. Such questions remain open and obviously await further studies.

## Supplementary information

Supplementary Material.

Dataset 1.
