# Supplementary material for: Insights from Microbial Transition State Theory on Monod’s Affinity Constant
Source: Sci Rep. 2020 Mar 24;10:5323. doi: 10.1038/s41598-020-62213-6 (PMC7093443; doi:10.1038/s41598-020-62213-6)
Supplement: Supplementary file 1 — Supplementary Material. [file 41598_2020_62213_MOESM1_ESM.pdf]

Supplementary Material
Insights from Microbial Transition State Theory on Monod's Affinity Constant

Pablo Ugalde-Salas[1] Elie Desmond-Le Quéméner[1] Jérôme Harmand[1]
Alain Rapaport[2] Théodore Bouchez[3]
[1]LBE, INRAE, Univ Montpellier, Narbonne, France
[2] MISTEA, INRAE, Univ. Montpellier, Montpellier SupAgro
[3] INRAE, UR PROSE, Antony, Centre d'Antony, Antony, France

Approximation

Consider the first order approximation of the exponential function:

$$\exp(x) = 1 + x + o(x) \tag{1}$$

and the first order approximation:

$$\frac{1}{1+x} = 1 - x + o(x) \tag{2}$$

Then rewrite MTS expression as follows:

$$\begin{aligned} \mu_{max} exp\left(\frac{-\lambda}{V_h s}\right) &\approx \mu_{max}\left(1 - \frac{\lambda}{V_h s}\right) & (3) \\ &\approx \mu_{max}\frac{1}{1 + \dfrac{\lambda}{V_h s}} & (4) \\ &= \mu_{max}\frac{s}{s + \dfrac{\lambda}{V_h}} & (5) \end{aligned}$$

Comparison of both expressions

The substrate limitation range can be studied through the ratio of both growth functions, shown in expressions (6) and (7), respectively.

$$\mu_{max}\frac{s}{s + \dfrac{\lambda}{V_h}} \tag{6}$$

$$\mu_{max} exp\left(\frac{-\lambda}{V_h s}\right) \tag{7}$$

Noting $K_s := \dfrac{\lambda}{V_h}$ One then considers the ratio:

$$R(s) = \frac{\exp\left(\frac{-K_s}{s}\right)}{\dfrac{s}{s + K_s}} \tag{8}$$

Note that ratio (8) does not depend on μ_{max}, It can be shown that $R(s) \in (0, 1)$, by using the well known inequality $\exp(x) < \frac{1}{1-x}$ for $x < 1$. Which is valid since the term inside the exponential is negative:

$$R(s) \leq \frac{1}{1 - \frac{-K_s}{s}} \frac{K_s + s}{s} = 1 \tag{9}$$

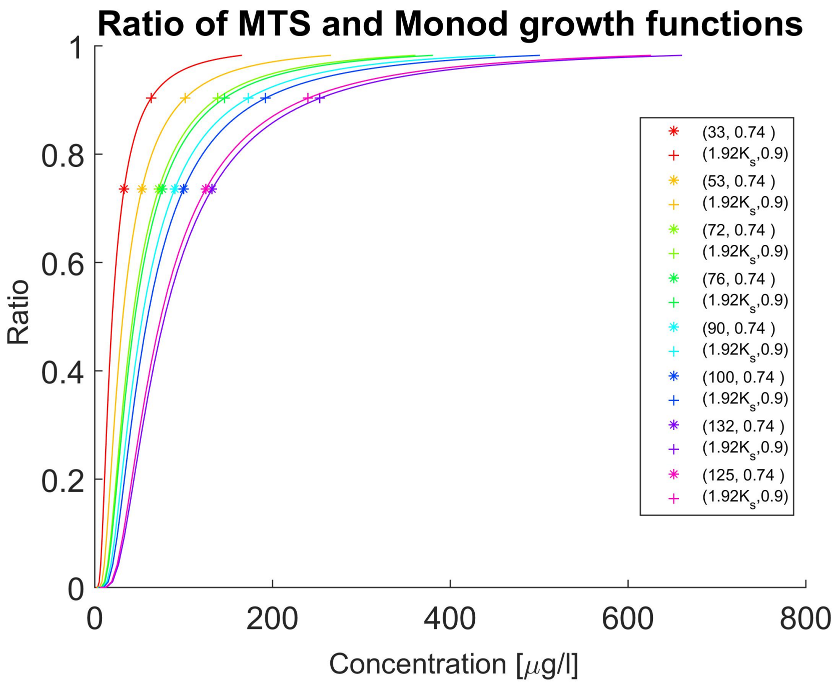

Figure 1: $R(s)$ curve for several K_s values obtained for E. Coli ML 30. The asterisk $*$ represents K_s values evaluated in expression $R(s)$, note that $R(K_s) \approx 0.74$. The plus sign $+$ represents the point where the $R(s) = 0.9$.

The change of variables $u = \frac{s}{K_s}$ is used to analyse the expression $R(s)$, which gives equation (10).

$$F(u) = \exp\left(-\frac{1}{u}\right)\frac{u+1}{u} \tag{10}$$

$$F'(u) = \exp\left(-\frac{1}{u}\right)\frac{1}{u^2}\frac{u}{u+1} + \exp\left(-\frac{1}{u}\right)\frac{1}{(u+1)^2} > 0 \tag{11}$$

Since F is monotonic, one gets that there exists a unique u^* such that $\exp\left(-\frac{1}{u^*}\right)\dfrac{u^*+1}{u^*} = 0.9$, implying a unique $s^* := u^* K_s$, such that $R(s^*) = 0.9$. From the former it can be seen that for each K_s MTS expression approximates to 90 % of the Monod expression whenever $s \geq u^* K_s$.

The curve $s \mapsto R(s)$ is shown in figure 1, for different K_s values of table 1 of the manuscript. One can see that $u^* \approx 1.92$ therefore $s \geq 1.92K_s$ then $R(s) \geq 0.9$.
